# p-Type Schottky Contacts for Graphene Adjustable-Barrier Phototransistors

**DOI:** 10.3390/nano14131140

**Published:** 2024-07-02

**Authors:** Carsten Strobel, Carlos Alvarado Chavarin, Martin Knaut, Matthias Albert, André Heinzig, Likhith Gummadi, Christian Wenger, Thomas Mikolajick

**Affiliations:** 1 Institute of Semiconductors and Microsystems, Chair of Nanoelectronics, Technische Universität Dresden, Nöthnitzer Straße 64, 01187 Dresden, Germany; martin.knaut@tu-dresden.de (M.K.); matthias.albert@tu-dresden.de (M.A.); andre.heinzig@tu-dresden.de (A.H.); likhith.gummadi@mailbox.tu-dresden.de (L.G.); thomas.mikolajick@tu-dresden.de (T.M.); 2 IHP—Leibniz-Institut für Innovative Mikroelektronik, Im Technologiepark 25, 15236 Frankfurt (Oder), Germany; alvarado@ihp-microelectronics.com (C.A.C.); wenger@ihp-microelectronics.com (C.W.); 3 Semiconductor Materials, Brandenburg University of Technology Cottbus-Senftenberg, Platz der Deutschen Einheit 1, 03046 Cottbus, Germany

**Keywords:** phototransistor, GABT, graphene, p-type, silicon, germanium, high responsivity, high speed, dual-band, photodetection

## Abstract

The graphene adjustable-barriers phototransistor is an attractive novel device for potential high speed and high responsivity dual-band photodetection. In this device, graphene is embedded between the semiconductors silicon and germanium. Both n-type and p-type Schottky contacts between graphene and the semiconductors are required for this device. While n-type Schottky contacts are widely investigated, reports about p-type Schottky contacts between graphene and the two involved semiconductors are scarce. In this study, we demonstrate a p-type Schottky contact between graphene and p-germanium. A clear rectification with on–off ratios of close to 10^3^ (±5 V) and a distinct photoresponse at telecommunication wavelengths in the infrared are achieved. Further, p-type silicon is transferred to or deposited on graphene, and we also observe rectification and photoresponse in the visible range for some of these p-type Schottky junctions. These results are an important step toward the realization of functional graphene adjustable-barrier phototransistors.

## 1. Introduction

High-speed photodetectors are a key building block in fiber optic communication systems, which primarily operate in the wavelength range from 1.3 to 1.55 µm [1]. Such devices are the main components in several applications such as long-haul data transition, local area networks, board-to-board, chip-to-chip, and intrachip interconnects. Photodiodes currently dominate the field of high-speed photodetection in the infrared wavelength range at telecommunication standards. Bandwidths of 170 GHz and responsivities of 0.27 A W^−1^ have been demonstrated with indium phosphide (InP) based photodiodes [2]. In 2021, the ultimate and benchmark photodetector performance was achieved with a p-i-n photodiode based on epitaxially grown germanium. A 3-dB bandwidth of 265 GHz and a responsivity of 0.3 A W^−1^ was verified [3]. Graphene (Gr) has also been employed for ultra-high speed (500 GHz) and tunable photodetectors but with moderate responsivity [4]. Another special feature of optical devices made of graphene is their potential absorption up to the THz range through surface plasmon resonance [5]. Novel in-fiber graphene-based devices with increased responsivities have also been reported [6]. Although today’s phototransistors have higher sensitivity than photodiodes, their speed is lower. For example, a responsivity of 10^7^ A W^−1^ has been demonstrated with a graphene-based phototransistor utilizing PbS quantum dots [7]. However, the bandwidth of this phototransistor is only 100 Hz. The development of photodetectors with high responsivity and high speed in the visible and short-wave infrared (SWIR) persists as a major challenge in optoelectronics. To tackle this challenge, a novel device called a graphene adjustable-barriers transistor (GABT) was proposed in 2022 [8]. The device can best be used as a high-performance dual-band phototransistor (photo-GABT) with sensitivity in the infrared and visible spectral range [9]. In this application scenario, ultra-high responsivities above 10^8^ A W^−1^ and a high speed with cutoff frequencies of more than 1 GHz are predicted [9]. Two device designs are conceivable. The first is an n-Si/Gr/p-Ge structure, while the second is a p-Si/Gr/n-Ge composition. As will be discussed later in this paper, the first design maximizes the device’s speed at telecommunication wavelengths in the infrared and is, therefore, called the IR configuration. In contrast, the second design allows for higher speeds at shorter wavelengths in the visible range and is, therefore, termed the VIS configuration. Although Gr/n-Ge [10,11] and n-Si/Gr junctions [12,13,14,15], which are part of the two photo-GABT designs, are widely examined, this is not the case for Gr/p-Ge and p-Si/Gr. Nonetheless, p-type Schottky junctions between Gr and p-Ge as well as Gr and p-Si are essential for the device operation of the various photo-GABT designs. For the first IR configuration with n-Si/Gr/p-Ge, the challenge is to fabricate a rectifying Gr/p-Ge junction. It is often argued that there could be no rectification at such a junction due to the orientation of the graphene and p-Ge work functions. This hypothesis seems to be confirmed by a study by Song et al. [16]. On the contrary, a significant p-type Schottky junction behavior was found even between n-Ge and graphene when Gr is grown directly on Ge [17]. This is attributed to the n- to p-type conversion of the entire Ge substrate due to the formation of a large density of acceptor defects during the graphene growth process. However, in this case, the area of the junction was in the nanometer scale, which limits the current density and application scenarios.

In the present study, we demonstrate for the first time a large area p-type Schottky junction between Gr and p-Ge with Gr transferred to the semiconductor. Rectification was observed for much larger device dimensions of up to 100 × 100 µm^2^. For the VIS configuration of the photo-GABT formed by a p-Si/Gr/n-Ge structure, a p-type Schottky junction between Gr and p-Si is required. While such p-type Schottky diodes were frequently implemented with Gr transferred on top of p-Si [18], investigations on devices with p-Si transferred or deposited on top of Gr are missing. This is also addressed in the present study. We will show I-V curves in the dark and under illumination for such silicon-on-graphene Schottky diodes. A clear rectification and photoresponse are observed for the selected p-Si/Gr devices. The Schottky barrier heights are deduced from I-V-T measurements. These results are an important milestone in realizing the first graphene adjustable-barrier phototransistor prototype devices.

## 2. Materials and Methods

Gallium-doped germanium wafers with a resistivity of 2–4 Ohm·cm (Siegert Wafer, Aachen, Germany) were used as the substrate for the Gr/p-Ge devices. The thickness of the p-Ge wafer is 500 µm. A cleaning procedure containing acetone, hydrogen peroxide, hydrofluoric acid, and RCA clean (Sigma Aldrich, Taufkirchen, Germany) was applied to reduce organic and metallic impurities from the wafer surface. Silicon dioxide was deposited by chemical vapor deposition with a thickness of 100 nm. An area of 50 × 50 µm^2^ was exposed from SiO_2_ by photolithography and subsequent hydrofluoric acid treatment (5%, 3 min). The 100 nm thick tungsten metallization was realized by photolithography, RF sputtering, and lift-off technique. Next, commercially available monolayer graphene (Graphenea, San Sebastian, Spain) was transferred on top of p-Ge by a poly(methyl methacrylate) (PMMA) assisted wet transfer approach. Another photolithography was applied to pattern Gr using an oxygen plasma treatment. Finally, titanium and aluminum were deposited on the backside of the substrate by electron beam evaporation. A picture of the fabricated devices is shown in Appendix A (Figure A2).

The p-Si/Gr test structure with the silicon transfer approach utilizes a silicon wafer (Siegert Wafer, Aachen, Germany) and a thermally grown SiO_2_ insulator. Tungsten and Gr were produced and patterned with photolithography similar to the p-Ge/Gr devices. To fabricate the transferred p-Si slice, a 500 µm thick, single-side polished monocrystalline silicon wafer (Siegert Wafer, Aachen, Germany) was covered with 10 nm of aluminum and 100 nm of ZnO:Al on the textured side of the wafer. The wafer was then annealed at 600 °C for 5 min to form an Al back surface field. Afterward, the wafer was cut by wire sawing to a size of 550 × 550 µm^2^ and transferred to the target location by means of micromanipulators. Thereby, the polished side of the p-Si slice points toward the underlying graphene.

The p-Si/Gr devices with p-Si deposited on Gr utilize a similar base technology for the framework (substrate + SiO_2_ + W + Gr) as described above. However, p-Si is sputter deposited from a polysilicon target (5N, Boron, 0.5 Ohm·cm, FHR Anlagenbau, Ottendorf-Okrilla, Germany). The fast annealing of p-Si takes place in an RTP tool at 1273 K with a heating rate of 2 K/s. The slow annealing is conducted in a furnace from ATV Technologie GmbH (Vaterstetten, Germany) at 1273 K with a heating rate of 10 K/min. The top contact consisted of Al/ZnO:Al and was brought up by photolithography.

The electrical characterization was conducted using a Keithley SCS 4200 semiconductor analyzer (Cleveland, Ohio, United States) connected to a vacuum probe station. To illuminate the devices with a wavelength of 450 nm, a mounted GaN-LED with 23 mW/cm^2^ maximum optical output power (Thorlabs GmbH, Bergkirchen, Germany) was used. The input power at the device was monitored by a PM400K5 optical power meter from Thorlabs GmbH. When illuminated with 1550 nm wavelength, a mounted LED with 260 mW/cm^2^ maximum optical output power was used.

## 3. Results and Discussion

### 3.1. Design Considerations of the Proposed Photo-GABT and Its Potential Performance

**Figure 1:**
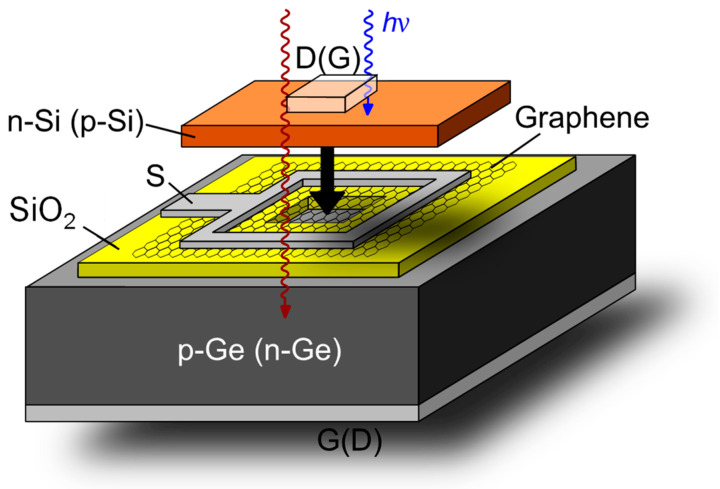
Structure of the photo-GABT’s IR configuration (VIS configuration) with a p-type (n-type) germanium bottom semiconductor and an n-type (p-type) silicon top semiconductor. S—source, D—drain, G—gate, hv—photon energy.

Figure 1 shows the three-dimensional device architecture of the novel photo-GABT device with the IR-(n-Si/Gr/p-Ge) and VIS configuration (p-Si/Gr/n-Ge). In the IR-design, graphene is embedded between an n-type silicon top semiconductor with an increased band gap of 1.1 eV and a p-type germanium bottom semiconductor with a low bandgap of 0.67 eV. The illumination takes place through the transparent conductive front contact, acting as drain electrode (D), and made of, e.g., ZnO:Al. A silicon dioxide layer is used to isolate the square-shaped source metallization from bulk germanium.

**Figure 2:**
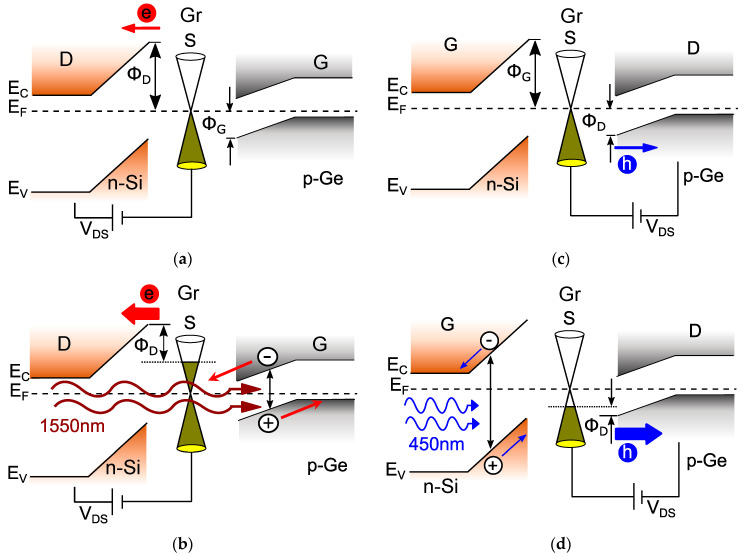
Simplified band diagrams of the photo-GABT operation states for different cases/conditions (**a**) SWIR operation in the OFF-state with the Schottky barriers Φ_G_ and Φ_D_. (**b**) ON-state with infrared illumination of 1550 nm wavelength. (**c**) VIS operation in the OFF-state with the Schottky barriers Φ_G_ and Φ_D_. (**d**) ON-state with illumination at 450 nm wavelength.

**Figure A1:**
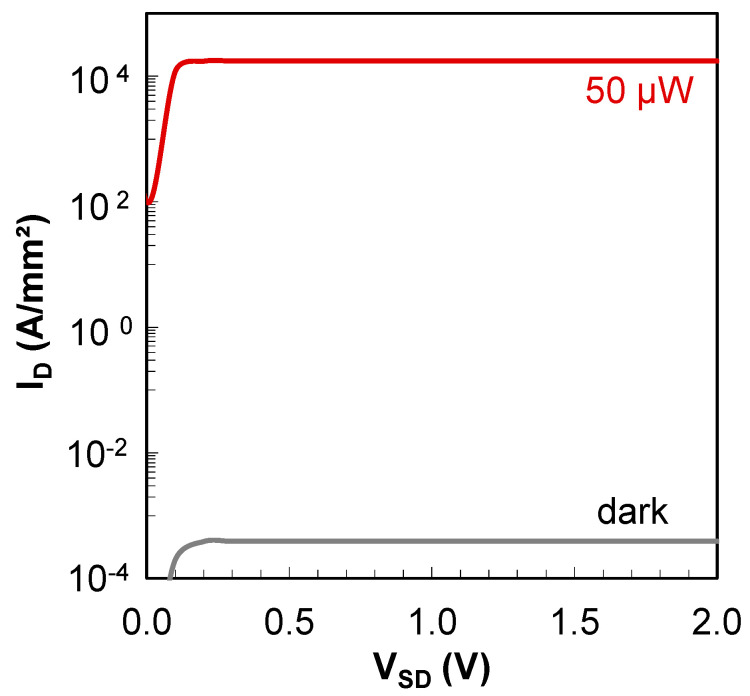
Calculated drain current density in the dark (grey curve) and with 50 µW illumination of 1550 nm (red curve).

**Figure A2:**
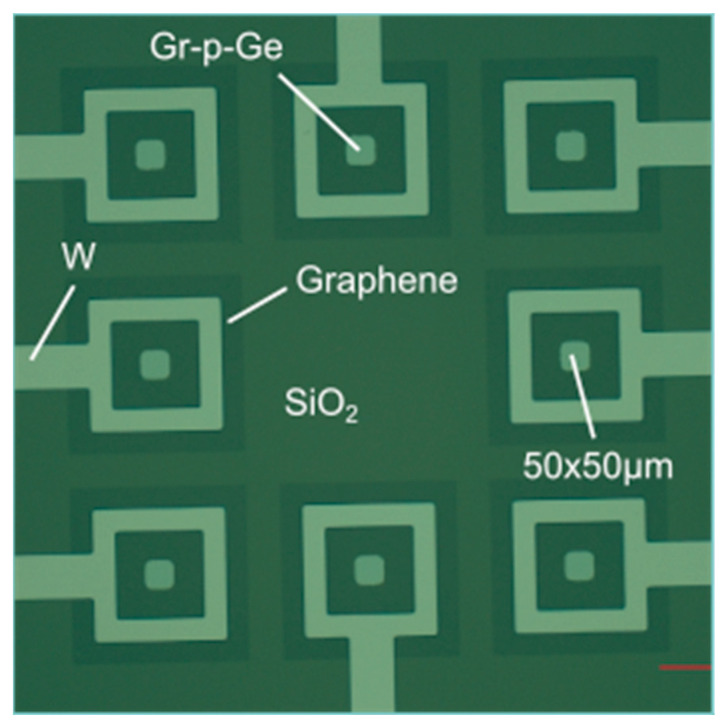
Optical microscope image of eight 50 × 50 µm^2^ Gr/p-Ge devices.

To explain the functionality of the new photo-GABT, we can look at the band diagram of the device (Figure 2). In this configuration, an inverse band bending with a Schottky barrier qΦ_G_ at the valence band forms between the gate semiconductor p-Ge and graphene (Figure 2a). Thus, this junction represents a p-type Schottky contact with barrier qΦ_G_, while the n-Si/Gr junction is an n-type Schottky contact with barrier qΦ_D_. In the OFF-state of the phototransistor, the large drain Schottky barrier qΦ_D_ restricts the current across the drain-source junction, as indicated by the small red arrow. Note that in this situation, the drain-source voltage V_DS_ itself is not capable of modulating the graphene Fermi energy level due to electrostatic screening by the thick depleted n-Si layer. This requires a drain semiconductor thickness larger than 300 nm. The ON-state (Figure 2b) is triggered by SWIR illumination. The light of, e.g., 1550 nm wavelength is transmitted through the n-Si with a wider bandgap and then absorbed in the p-Ge gate semiconductor with a smaller bandgap. Within the graphene/p-Ge space charge region, the absorbed light generates electron-hole pairs, which are separated by the potential gradient of the Schottky junction. Thus, electrons move toward and accumulate in graphene and also shift the graphene Fermi energy level toward the conduction band E_C_. This leads to a decreasing drain Schottky barrier qΦ_D_, and the current across the drain-source junction increases (big red arrow, Figure 2b). A similar shift of the Gr Fermi energy level and subsequent reduction in the drain Schottky barrier height, triggered by a gate voltage instead of illumination, was demonstrated for the barristor device [19]. Calculations indicate a potentially ultra-high responsivity of 2.8 × 10^8^ AW^−1^ for the photo-GABT operation under SWIR illumination (see Appendix A, Figure A1). Furthermore, the potential speed of the device is calculated to be beyond 1 GHz (see Appendix A). Such a potentially high-speed and high-responsivity device is unique amongst today’s photodetection technologies.

Apart from the outstanding responsivity and speed, another potential feature of the photo-GABT is the possibility of dual-band operation independent of the configuration. To be specific, the photo-GABT can also operate in the visible spectral range (VIS, λ = 380–750 nm). For example, Figure 2c,d show the OFF- and ON-states of the photo-GABT under 450 nm illumination, respectively. In this case, the roles of the semiconductors are switched, and now n-Si acts as the gate, while p-Ge is the drain terminal. In the OFF-state without illumination (Figure 2c), the negatively biased drain features only a small hole current across the drain Schottky barrier Φ_D_, as indicated by the thin blue arrow. In the ON-state (Figure 2d), the blue light illumination of, e.g., 450 nm generates electron-hole pairs within the n-Si gate semiconductor. These electron-hole pairs are then separated in the n-Si/Gr space charge region, and the holes move toward graphene. The accumulation of holes in Gr decreases the Fermi energy level toward the valence band, and their drain Schottky barrier height qΦ_D_ is also lowered. Thus, a large current across this barrier is enabled, as indicated by the big blue arrow in Figure 2d. As the device operation in the VIS range relies on hole transport, exhibiting lower mobility, the speed of the photo-GABT might be slightly reduced compared to the operation in the SWIR range. If the maximum speed is required in the VIS range, the device structure can be inverted to the VIS configuration (p-Si/Gr/n-Ge), as already described in Ref. [9]. In this case, the challenge is the fabrication of p-type Schottky contacts between p-Si on top of graphene, while for the IR configuration (n-Si/Gr/p-Ge), p-type Schottky contacts for Gr on top of p-Ge are required. In the following section, initial results on p-type Schottky contacts between silicon, germanium, and graphene are presented.

### 3.2. p-Type Schottky Junctions for the Photo-GABT

#### 3.2.1. Graphene on p-Germanium

**Figure 3:**
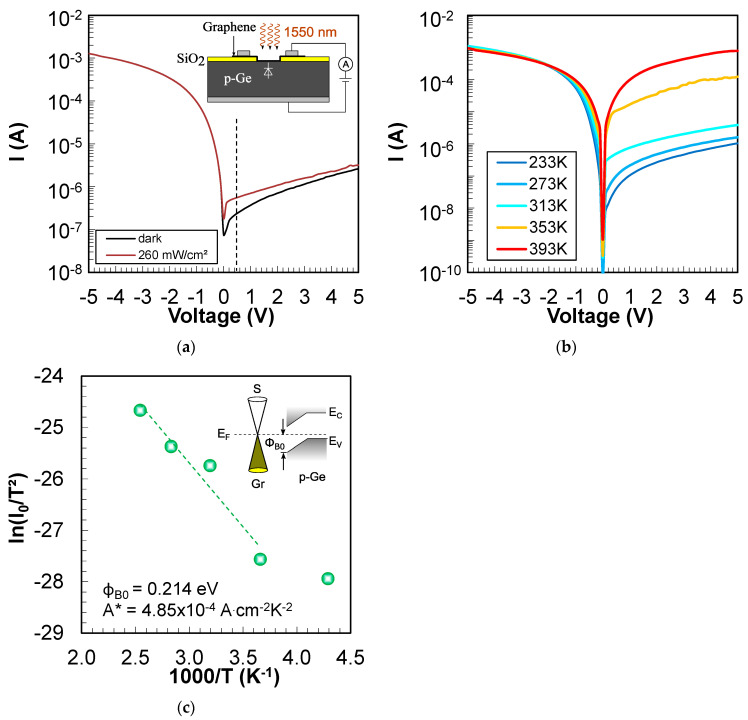
p-Type Schottky contact characterization of graphene on p-Ge (**a**) I-V curve of the Gr/p-Ge Schottky junction in the dark (black curve) and under illumination with a wavelength of 1550 nm (brown curve), dashed line represents the reference point for net photocurrent calculation, inset: biasing of the junction. (**b**) I-V-T plot of the Gr/p-Ge junction measured in the dark. (**c**) Richardson plot of the Gr/p-Ge junction with the extracted Schottky barrier height Φ_B0_ and Richardson constant A*.

The I-V plot of the Gr/p-Ge junction in the dark and under illumination with a wavelength of 1550 nm is shown in Figure 3a. The black curve (dark I-V) reveals a clear rectification with an on–off ratio at ±5 V of approximately 5 × 10^2^ (±2.9 × 10^2^). The biasing of the junction is illustrated in the inset of Figure 3a. Together with the measurement, this verifies the p-type Schottky contact behavior. When illuminated with a wavelength of 1550 nm, a distinct photoresponse under the reverse bias of the Gr/p-Ge diode can be observed. The responsivity of this non-optimized diode is approximately 1 × 10^−4^ A W^−1^. The I-V curves were also measured as a function of temperature (see Figure 3b) in order to extract the Schottky barrier height. Thereby, the diode current of a p-type Schottky diode is described by [17]:

(1) $$I=-I_{0}\left(e^{-\frac{q\left(V-R_{s}I\right)}{nkT}}-1\right)\;$$

where I is the current, q is the elementary charge, V is the voltage applied to graphene, $R_{s}$ is the series resistance, n is the diode ideality factor, k is the Boltzmann constant, T is the temperature, and $I_{0}$ is given by the following equation:

(2) $$I_{0}=AA^{*}T^{2}e^{-\Phi_{B0}/kT}$$

where A, $A^{*}$, and $\Phi_{B0}$ represent the junction area, the Richardson constant of the semiconductor, and the Schottky barrier height, respectively. A conventional Richardson plot can be used to evaluate the Schottky barrier height by taking the natural logarithm of Equation (2):

(3) $$ln\left(\frac{I_{0}}{T^{2}}\right)=ln\left(AA^{*}\right)-\frac{q\Phi_{B0}}{kT}\;$$

Figure 3c shows the Richardson plot of $\ln\left(I_{0}/T^{2}\right)$ versus 1000/T. The barrier height of the Gr/p-Ge junction is deduced from a linear fit to the data and represents 0.214 eV (±0.02 eV). This barrier height is comparable to Gr/n-Ge junctions where values between 0.2–0.3 eV have been reported [8,11]. The inset in Figure 3c illustrates the band bending at the Gr/p-Ge junction with the Schottky barrier $\Phi_{B0}$ toward the valence band. The Richardson constant is calculated to 4.85 × 10^−4^ A·cm^−2^·K^−2^ which is consistent with previous reports about Gr/n-Ge junctions [20]. The $\ln\left(I_{0}/T^{2}\right)$—1000/T plot deviates from linearity at very low temperatures which indicates a strong temperature dependence of the barrier height. A decreased Schottky barrier at very low temperatures could also affect the performance of the photo-GABT. However, as the targeted photo-GABT device should perform well at increased temperatures (i.e., 293 K), the deviation from linearity at very low temperatures can be neglected.

The net photocurrent of the Gr/p-Ge diode at a wavelength of 1550 nm is $I_{net}=I_{photo}-I_{dark}$ and represents 3.21 × 10^−7^ A at a voltage of +0.5 V (see dashed line in Figure 3a). This means that about 2 × 10^9^ electrons are accumulating in Gr on the timescale of a millisecond. This translates to a change in the Fermi energy level in Gr of 0.052 eV, and at the drain of the GABT phototransistor with an n-Si top semiconductor, the current would increase by about one order of magnitude. However, to fully turn on the photo-GABT, further improvements are required to increase the photocurrent at the Gr/p-Ge junction. This could be achieved by, e.g., passivation of the p-Ge surfaces and implementation of an aluminum-based back surface field at the p-Ge back contact [21].

#### 3.2.2. p-Silicon on Graphene

*(A) Transfer method*

**Figure 4:**
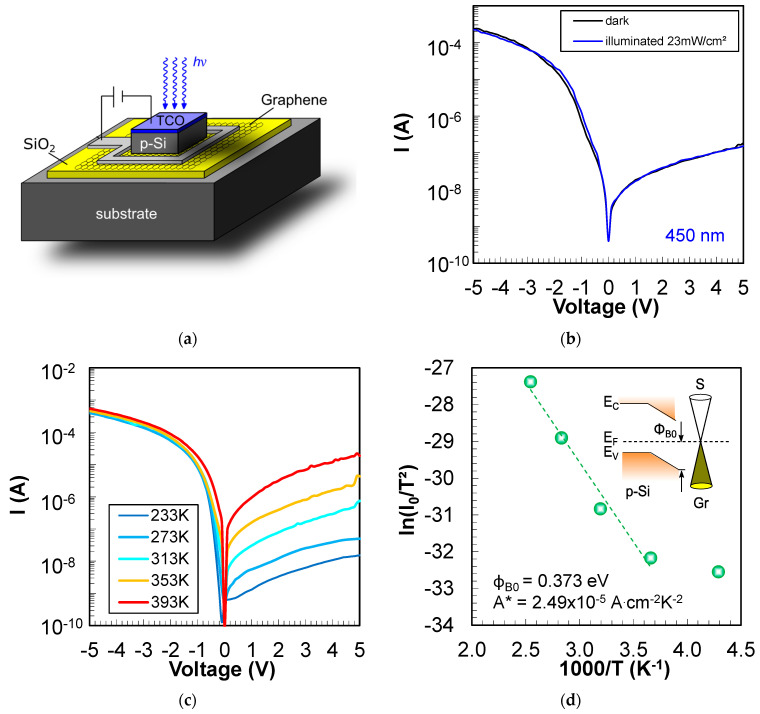
Investigation of the p-Si/Gr junction utilized for the VIS configuration of the photo-GABT. (**a**) Test structure for the evaluation of the p-Si/Gr p-type Schottky contact. (**b**) I-V curves of the p-Si/Gr junction in the dark and under illumination. (**c**) I-V-T plot of the p-Si/Gr junction measured in the dark. (**d**) Richardson plot of the p-Si/Gr junction with the extracted Schottky barrier height Φ_B0_ and Richardson constant A*.

As a first attempt to investigate the p-Si/Gr Schottky contact for the VIS configuration of the photo-GABT, 550 µm thick monocrystalline p-silicon is transferred onto Gr (see methods section). Figure 4a shows the resulting test structure and biasing scheme with p-Si illuminated through a transparent conductive oxide (TCO—here: ZnO:Al). Thereby, graphene is insulated from the substrate through a SiO_2_ layer. The I-V curves in the dark and under illumination are illustrated in Figure 4b.

**Figure A3:**
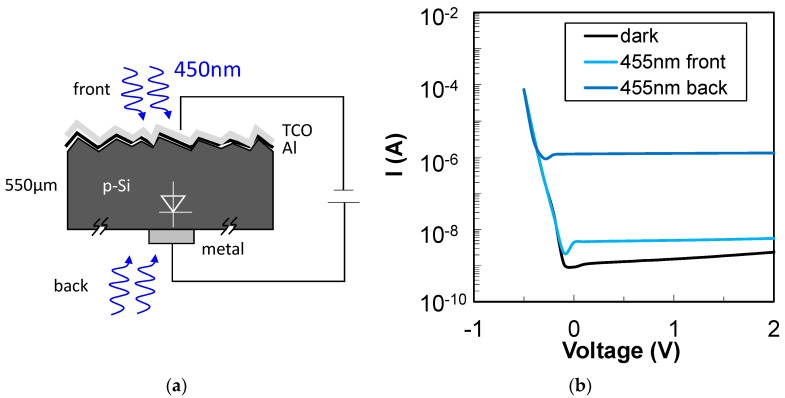
p-Si/metal reference device under front and backside illumination (**a**) Cross section of a test structure and the measurement setup for the evaluation of front and back side illumination. (**b**) I-V curves of the p-Si/metal test structure in the dark (black curve), under front illumination (light blue curve), and under back side illumination (dark blue curve).

A distinctive rectification with an on–off ratio of 1.2 × 10^3^ (mean 5.7 × 10^2^ at ±5 V) could be observed for the p-type Schottky contact between p-Si and Gr. Unfortunately, the current under illumination is the same as in the dark, which means that no significant photoresponse could be verified for this device. To better understand the lack of a photocurrent for the p-Si/Gr device, a reference p-Si/metal structure was fabricated and illuminated from the front side and from the back side (see Appendix B, Figure A3). This reference test structure reveals a much stronger photoresponse when illuminated from the back side. When illuminated through the front side with a short wavelength of 450 nm, most of the carriers are absorbed near the surface of the wafer. This indicates that there is a problem with the diffusion of carriers from the front side to the back side of the wafer in case of front side illumination. However, at least a slight photoresponse could be expected for the p-Si/Gr junction under front side illumination as observed for the reference metal/p-Si device (see Figure A3b). This slight photoresponse shown in Figure A3b also demonstrates that the diffusion length in our p-Si wafers is higher than the wafer thickness of 500 µm. It can be speculated that an increased recombination at the wire-sawed surfaces of the transferred p-Si slice impedes any photoeffect for the p-Si/Gr device. The as-cut wafer’s front side could also be a source of high recombination, especially when considering that most of the blue light is absorbed near the surface. The boron-oxygen complex [22] could also lead to high recombination in p-Si, which could substantially reduce photoresponse. Novel p-Si transfer techniques based on silicon-on-insulator (SOI) wafers [11] with strongly reduced p-Si thicknesses and improved p-Si membrane surfaces might be suitable to strongly increase the photoresponse of future devices.

The p-Si/Gr Schottky junction was also measured at different temperatures (Figure 4c) to extract the Schottky barrier height from the Richardson plot (Figure 4d). A Schottky barrier height of 0.37 eV (mean 0.3 eV) was drawn for the transferred p-Si/Gr junction. In the literature, Schottky barrier heights between 0.18 eV and 0.47 eV for graphene transferred on top of p-silicon have been reported [18,23,24]. Thus, with the present work, similar barrier heights could be demonstrated for the first time for p-Si transferred on top of Gr. The inset of Figure 4d shows the band bending at the p-Si/Gr junction which fulfills the requirements for the photo-GABT.

*(B) Deposition of p-Si*

**Figure 5:**
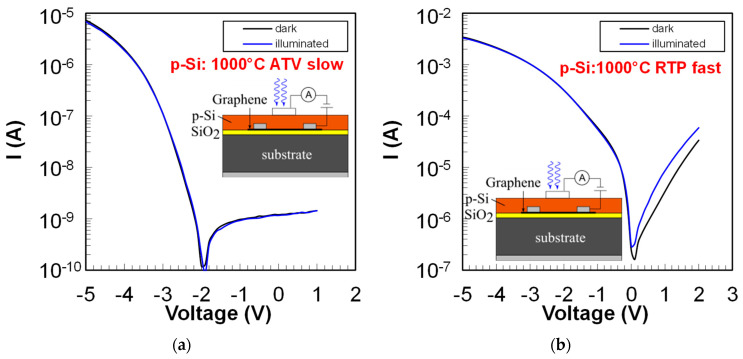
p-Si to Gr contact characterization for different p-Si deposition techniques (**a**) I-V curves of the p-Si/Gr junction in the dark and under illumination for p-Si annealed with slow heating rates at 1000 °C in an ATV furnace. (**b**) I-V curves of the p-Si/Gr junction in the dark and under illumination for p-Si annealed with fast heating rates at 1000 °C in an RTP furnace.

The second, more scalable approach to investigate the p-Si/Gr Schottky contact for the VIS configuration (p-Si/Gr/n-Ge) of the photo-GABT is to deposit and crystallize p-type amorphous silicon (p-a-Si) on top of graphene (see methods section). Thereby, p-a-Si is deposited by RF-sputtering and crystallized through high temperature annealing at 1000 °C. Moreover, the annealing was either executed with a slow heating rate in a furnace from ATV Technologie GmbH or with fast ramps in a rapid thermal processing (RTP) tool. Figure 5 shows the I-V curves of p-Si deposited and crystallized on top of graphene. As can be seen, the I-V curves for the slow annealing process (Figure 5a) show a distinct rectification with a p-type Schottky contact behavior between p-Si and Gr.

**Figure A4:**
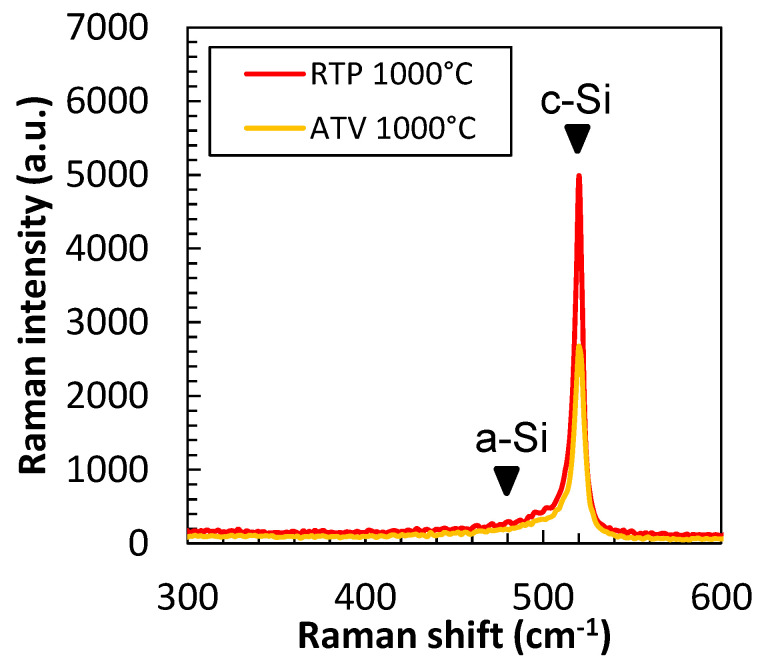
Raman spectrum of sputter deposited p-Si annealed at 1000 °C with a rapid thermal processing (RTP) treatment (red curve) or with a slow process in an ATV furnace (orange curve).

However, a photoresponse in the reverse direction (blue vs. black curve in Figure 5a), as required for the p-Si/Gr/n-Ge photo-GABT, could not be observed. Furthermore, even if the reverse currently shows saturation, the I-V curve does not pass through the origin, and the forward current is rather low. In contrast, the forward current for the fast RTP anneal (Figure 5b) is much higher, and a distinct photoresponse in the reverse direction can be observed. The shape of the curve also indicates a p-type Schottky contact between p-Si and Gr. However, the reverse current of the RTP anneal curve shows no saturation so far. The reason for the observed photoresponse in the case of the fast annealing could be an increased crystalline volume fraction (see Raman spectra in Appendix B, Figure A4) for the RTP material. It can be speculated that different crystallization dynamics lead to an increased crystallinity for the material annealed with fast heating rates. Although currently, the fast annealing seems more promising toward a functional p-Si/Gr/n-Ge photo-GABT, further optimizations are required to improve the p-Si/Gr junction.

### 3.3. Comparison of Phototransistor Performances

Silicon bipolar phototransistors for the visible range are readily available on the market. These Si npn phototransistors are comparable to those presented more than 20 years ago by Dalla Betta et al., reaching responsivities close to 0.6 A/W at wavelengths of ~850 nm, falling close to zero at ~1000 nm and showing a 3 dB bandwidth of 55 kHz [25]. In the short-wavelength infrared, outstanding results have been shown recently for Si/Ge waveguide phototransistors by Gao et al. [26], reaching responsivities at over 1000 A/W and a 3 dB bandwidth of 1.5 GHz for a wavelength of 1550 nm. It can be noted that even with such high responsivities, waveguide-based photodetection still requires light to be coupled to the waveguide, with, e.g., a grating coupler, thus increasing the overall insertion losses of this strategy. To improve the spectral range, responsivity, and bandwidth, other materials have been investigated. Son Ko et al. [27] used InP Nanopillars directly grown on Si, reporting a responsivity of 9.5 A/W and a bandwidth of 7 GHz for a 785 nm wavelength. Han et al. [28] used a combination of organic semiconductor crystals and nanoparticles to achieve a broadband responsivity over 1.2 × 10^5^ A/W from 410 to 740 nm in wavelength. Besides the CMOS incompatibility of organic semiconductors, the response times are usually in the ms range. Thus, very low bandwidths are expected. 2D materials have also been investigated as phototransistors. Xu et al. [29] reported a responsivity of ~2.5 × 10^4^ A/W for dual gate phototransistors based on WSe_2_. Moudgil et al. [30] used In_2_S_3_ on Si, achieving a responsivity of 12 A/W and 41 A/W for illumination at 405 nm and 800 nm wavelengths, respectively. Using black phosphorous (BP) on Si-doped Ga_2_O_3_, Chen et al. [31] reported a responsivity of 2390 A/W and 0.53 A/W under 254 nm and 808 nm illumination, respectively. A p-WSe_2_/n-Ge heterojunction transistor was investigated by Li et al. [32], which achieves responsivities of 55 A/W, 95 A/W, and 120 A/W at illuminations of 405 nm, 1310 nm, and 1550 nm, respectively. The aforementioned 2D approaches mainly used exfoliated sheets, which are known to lack upscaling fabrication capabilities. Furthermore, the reported response times range from µs to s, and thus, limited to very limited bandwidths are expected. Further information on photodetection based on graphene transistors can be found elsewhere [33]. In the specific case of the dual barrier Si/Gr/Ge phototransistor (photoGABT) approach, a plausible advantage is the possibility of the direct growth of Gr on Ge reported by several research groups [34,35,36]. In addition, analytical calculations (see Appendix A) show that bandwidths in the GHz range could be expected. A comparative table of selected phototransistors is shown in Table 1.

## 4. Conclusions

As a prerequisite for the novel, potentially high-performance graphene adjustable-barrier phototransistor, p-type Schottky contacts between graphene and p-Ge or p-Si are required. In this study, we demonstrate a rectifying p-Ge/Gr p-type Schottky junction with a significant photoresponse at telecommunication wavelengths of 1550 nm, which is against theoretical expectations. The Schottky barrier height for the Gr/p-Ge diode is determined to be 0.214 eV. Furthermore, the transfer and deposition of p-Si on top of graphene have also been investigated. We found a rectifying behavior for all studied p-Si/Gr test structures. The mean Schottky barrier height for the transferred p-Si on Gr devices is 0.3 eV. A distinct photoresponse at a wavelength of 450 nm is observed for the p-Si/Gr junction when p-Si is deposited and crystallized at 1000 °C in a rapid thermal processing tool. These results pave the way for the realization of first functional graphene adjustable-barrier phototransistor devices.

## 5. Patents

C. Strobel, “Halbleiterbauelement, Verfahren zum Herstellen eines Halbleiterbauelements und Verfahren zum Betreiben eines Halbleiterbauelements”, DPMA P83689, vol. Patent 10 2022 106 012.8 DE, Mar. 2023.

C. Strobel, “Photodetektor und Verfahren zum Betreiben eines Photodetektors”, DPMA P87474, vol. Patent 10 2023 113 982.7 DE, May 2023.

## Figures and Tables

**Table 1:** Comparison of phototransistor performances for various transistor architectures. Last row is marked in grey and represents “proposed in this work”.

Ref.	Phototransistor Type	Responsivity (AW^−1^)	Spectral Range	Bandwidth	CMOS Compatibility
[25]	Si npn	0.6 (at 850 nm)	Broadband (400–1000 nm)	55 kHz	High
[26]	SiGe waveguide	1032	IR (1550 nm)	1.5 GHz	High
[27]	InP nanopillars on Si	9.5	Visible (785 nm)	7 GHz	Medium
[28]	Organic semiconductors	1.2 × 10^5^	Broadband (410–740 nm)	Limited	Low
[29]	WSe_2_ on hBN	2.5 × 10^4^	Visible (532 nm)	Limited	Low
[30]	In_2_Se_3_ on Si	41 (at 800 nm)	Broadband (405–800 nm)	Limited	Low
[31]	BP on Ga_2_O_3_	2390 (at 254 nm)	Broandband (254–808 nm)	Limited	Low
[32]	WSe_2_ on Ge	120 (at 1550 nm)	Broadband (405–1550 nm)	Limited	Low
	Double barrier Si/Gr/Ge	2.8 × 10^8^	Broadband (532–1550 nm)	25 GHz	Medium

## Data Availability

Data are contained within the article.

## Appendix A

*Calculation of main photo-GABT device metrics*

The calculated output currents of the new n-Si/Gr/p-Ge photo-GABT in the dark and under 1550 nm illumination (50 µW) are shown in Figure A1. High output currents above 10^4^ A/mm^2^ are achieved under SWIR illumination. The high output current density is derived from the following considerations. The number of photons from a SWIR laser (1550 nm, 50 µW, 1 mm^2^, 1 ms) impinging on the phototransistor can be calculated as 3.9 × 10^11^. Assuming a moderate external quantum efficiency of 37%, this results in about 1.4 × 10^11^ electrons moving to the graphene. The charge carrier-induced increase in the Fermi level in graphene can be calculated to 0.44 eV using the relationship $\Delta E_{F}=\hbar v_{F}\sqrt{\pi n}$ [19] with the Dirac constant ℏ, the Fermi velocity of graphene $v_{F}$ and the charge carrier density n. Furthermore, in the initial state the graphene/n-silicon Schottky barrier height is 0.49 eV [12] and then decreases to 0.05 eV due to the SWIR illumination. If one now takes the diode equation for the thermionic emission via the Schottky barrier with qΦ_D_ = 0.05 eV:

(A1) $$J=A^{**}T^{2}exp\left(-\frac{q\phi_{D}}{kT}\right)\left[exp\left(\frac{qV_{DS}}{nkT}\right)-1\right]\;$$

With $A^{**}$ being the Richardson constant (120 A cm^−2^ K^−2^), T being the temperature, and k being the Boltzmann constant. Then, a reverse saturation current density of 1.4 × 10^4^ A/mm^2^ is calculated. This corresponds to the illumination-induced ON-current as observed in Figure A1 (red curve). Currents of this magnitude are indeed predicted for germanium-based GABTs [8]. This means that about 1 × 10^20^ electrons per millisecond flow across the drain Schottky barrier, and, based on the incoming photons, there is an ultra-high gain of about 2.5 × 10^8^ and a responsivity of 2.8 × 10^8^ A W^−1^ at this operating point. This would be amongst the highest reported gains of photodetectors.

Moreover, the speed of such a Schottky barrier-based phototransistor is determined by the transit time of the carriers through the depletion layer, the sheet resistances, the resistance of the bulk material, and the capacitance of the junction. The electron transit time $\tau_{el}$ can be calculated from the relation [37]: $\tau_{el}=d/v_{s}$, with d the thickness of the space charge region, and $v_{s}$ the saturation velocity. The latter is 1 × 10^7^ cm/s and 0.6 × 10^7^ cm/s for electrons in silicon and germanium, respectively. In order to absorb about 40% of light in p-Ge (see required EQE), a minimum p-Ge thickness of about 1 µm can be derived from Lambert–Beer’s law. The calculated space charge region thickness of a Gr/p-Ge junction is also about 1 µm if a slightly negative voltage of about −0.5 V is applied to the gate. In this case, the device operation only relies on a fast drift process as no carriers must slowly diffuse toward the depletion region. In addition, as already discussed above, the minimum n-Si thickness corresponds to 0.3 µm. Thus, a good approximation for the minimum required space charge region thickness an electron must travel in total through the device is d=1.3 µm. This yields a $\tau_{el}\sim 20\;\mathrm{ps}$ and a cutoff frequency of 51 GHz. Furthermore, based on the capacitance C per unit area and the sheet resistance $R_{s}$, the frequency response of a Schottky detector can be calculated [37]:

(A2) $$w_{c}=\frac{3}{R_{s}Cb^{2}}\;$$

with $w_{c}$ being the 3-db cutoff frequency and b being the width of the device. With a capacitance of about 7.5 × 10^−3^ F/m^2^ [38] (area 4 × 10^−4^ cm^2^), a total resistance of 50 Ohm, and a device width of 10 µm, a cutoff frequency $f_{c}=w_{c}/2\pi$ ≈25 GHz seems feasible. This cutoff frequency is of the same order as the cutoff frequency obtained from transit time considerations. Thus, the photo-GABT potentially offers a very high bandwidth.

*Image of the Gr/p-Ge devices*

In Figure A2, the top view of eight individual Gr/p-Ge devices is illustrated. We use a 100 nm thick underlying SiO_2_ layer to realize a good optical contrast in order to visualize the graphene layer in the optical microscope. Thereby, Gr is patterned to islands of 350 × 350 µm^2^ by O_2_-plasma.

## Appendix B

A reference device that resembles the p-Si/Gr structure (see Figure 4a) was fabricated. Figure A3 shows the design of this test structure. The 550 µm thick p-type silicon wafer is metalized from the back side through a shadow mask. The size of the metal contact is of the same order as the transferred p-Si flake of the device shown in Figure 4a). It can be assumed that the metal/p-Si contact (see Figure A3a) approximately resembles the semi-metallic graphene/p-Si contact of the test structure in Figure 4a. The front contact of the test structure consists of a full area Al/TCO deposit. Next, the p-Si/metal device is illuminated from the front side and from the back side via a 450 nm LED. The I-V curves of the p-Si/metal test structure are shown in Figure A3b. It can be seen that a strong photoresponse is present under back side illumination even though the metal contact is non-transparent. Thus, a strong lateral diffusion of photo-induced carriers must be available. However, under front side illumination, only a very low photoresponse could be observed. As most of the light of 455 nm wavelength is absorbed near the surface of the wafer, this indicates an insufficient diffusion of carriers from the front to the back side of the p-Si wafer.

*Raman measurement of sputter deposited and annealed p-Si*

The Raman spectra of sputter deposited and crystallized p-Si were measured for material annealed fast (RTP) and slowly (ATV) at 1000 °C. Figure A4 shows the corresponding curves with a distinctive peak at 520 cm^−1^, which can be attributed to the crystalline phase of p-Si. The Raman spectra demonstrate that both the materials that were annealed with fast (2 K/s) and slow (10 K/min) ramps were successfully crystallized. However, the Raman intensity for the RTP material is almost double as high as for the ATV material.

We attribute the increased Raman intensity to an increased crystalline volume fraction of the RTP material. This could be due to different crystallization dynamics when annealed with fast ramps compared to slow ramps.
